# Pasteurella Multocida Bacteremia and Septic Arthritis in a Patient With Non-Bite Animal Exposure

**DOI:** 10.7759/cureus.14162

**Published:** 2021-03-28

**Authors:** Lex P Leonhardt, Aamir Pervez, Wesley Tang

**Affiliations:** 1 Internal Medicine Residency, Kettering Medical Center, Dayton, USA

**Keywords:** pasteurella multocida, bacteremia, septic shock, septic arthritis, chronic wounds, diabetic foot ulcers, diabetic foot ulcers management

## Abstract

*Pasteurella multocida* (*P. multocida*) is a gram-negative, facultative anaerobe, and it is most commonly found in the oropharynx of healthy domestic animals, especially cats and dogs. *P. multocida* is classified as an opportunistic pathogen, infecting individuals only through direct contact via local trauma caused by animal bites or scratches. More serious infections, while rare, are typically associated with immunocompromised states, known pre-existing cavitary lung pathology, or malignancy. In this report, we present a case of *P. multocida* infection without known trauma, which had likely spread via respiratory droplets causing bacteremia, septic shock, and subsequent septic arthritis of the left knee.

## Introduction

*Pasteurella multocida* (*P. multocida*) is a ubiquitous gram-negative, facultative anaerobe, which is commonly found in the oropharynx of cats and dogs. In general, most human infections are the result of zoonotic transmission via cat/dog bites and/or scratches. Consequently, most infections where *P. multocida* has been isolated primarily involve the skin or soft tissue. Despite penetrating injury being the most common mechanism of infection, serious infections such as bacteremia, peritonitis, and meningitis are exceedingly rare. In more serious infections, the host typically has certain medical conditions such as pre-existing cavitary lung pathology, immunocompromised state, malignancy, or dialysis dependence.

In this report, we present the case of a patient with chronic lower extremity wounds who developed septic shock from *P. multocida* bacteremia and was subsequently found to have septic arthritis of the left knee. The patient did not have any known inciting bite or scratch. We postulate that respiratory secretions from the patient’s cats entered his wounds, and subsequently seeded the patient’s circulatory system leading to the clinical presentation of septic shock.

## Case presentation

**Figure 1:**
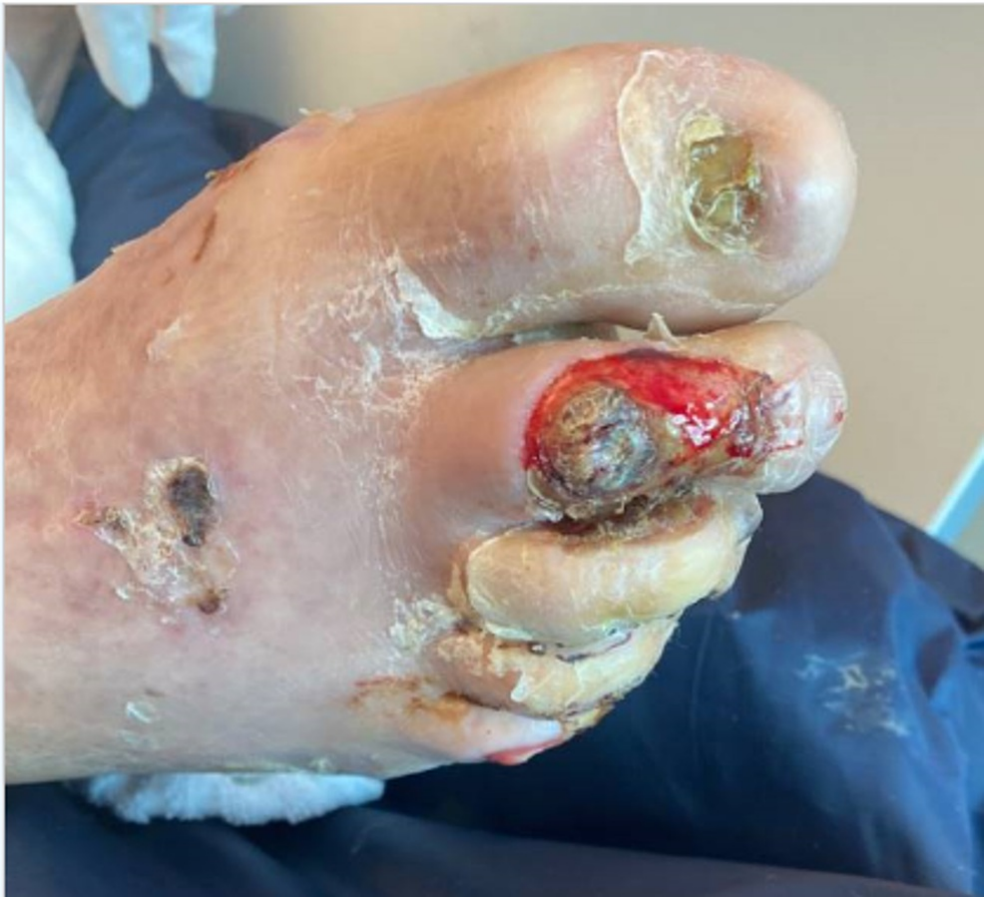
Ulceration of the right second toe

**Figure 2:**
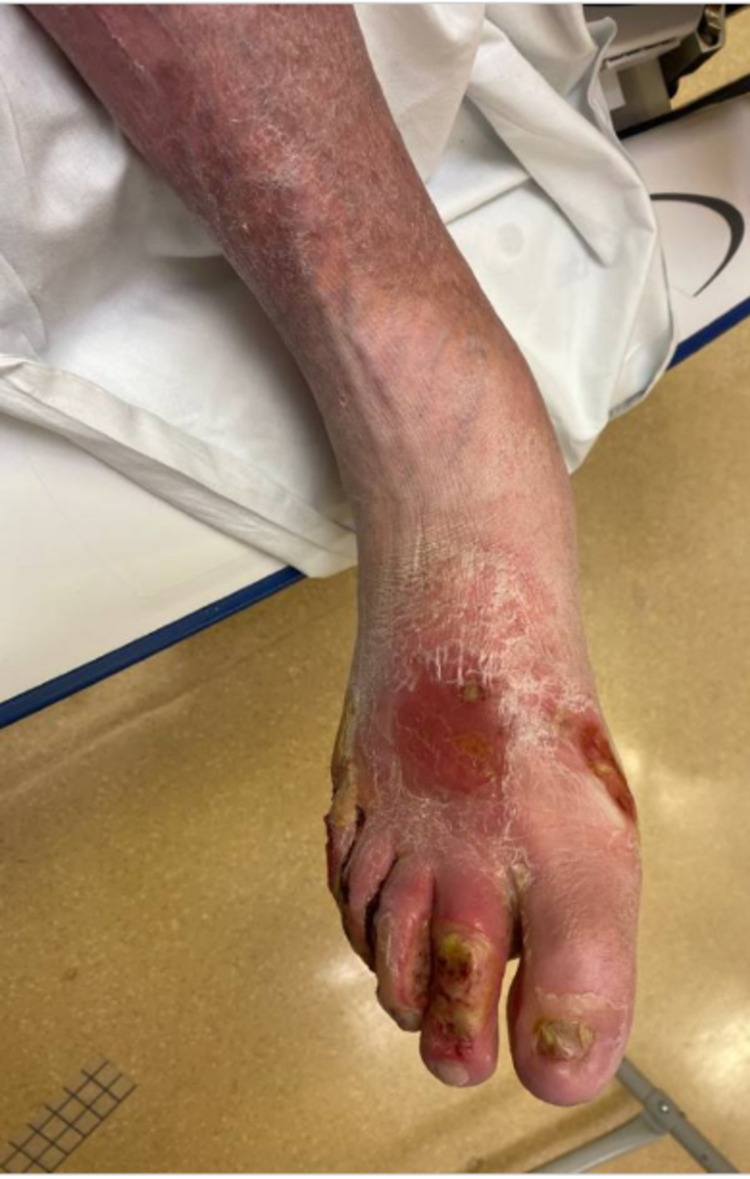
Chronic ulcerations over the dorsal aspect of the right foot

**Figure 3:**
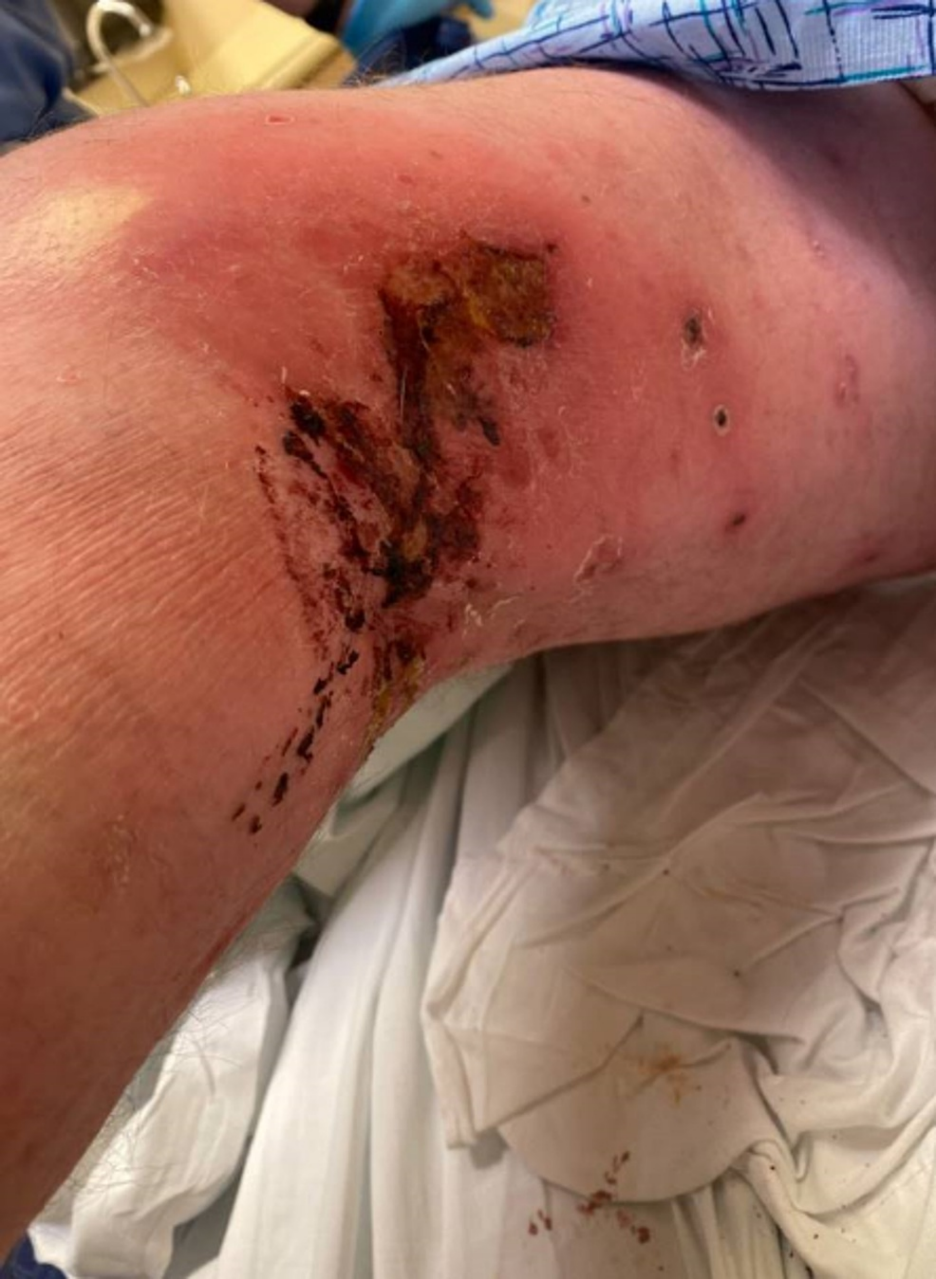
Posteromedial right knee ulceration

A 55-year-old male with a history of chronic systolic congestive heart failure, paroxysmal atrial fibrillation on Eliquis, and diabetes mellitus type 2 complicated by peripheral neuropathy and chronic diabetic foot ulcers with multiple other comorbidities presented to the emergency department complaining of bilateral knee and right shoulder pain after falling multiple times at home the previous day. Initial vital signs were notable for fever with Tmax of 100.8 °F, hypotension with a blood pressure of 70/40 mmHg, and atrial fibrillation with a rapid ventricular response, and a heart rate of 150 bpm. Physical exam revealed multiple ulcerations over bilateral lower extremities (Figures 1, 2, 3). Bilateral knee effusions were also present, left greater than right. Severe pain and crepitus were noted with both active and passive range of motion of the left knee. Laboratory workup was notable for WBC of 14,000 K/uL, creatinine of 1.7 g/dL (baseline: 1.3 g/dL), and initial lactic acid of 2.5 mmol/L. Two sets of blood cultures were collected from the left hand and left arm.

Intravenous (IV) vancomycin 1750 mg and Cefepime 2000 mg were administered after the collection of blood cultures in the emergency department. The patient reported a history of dizziness and rash caused by beta-lactam antibiotics. Aggressive intravenous fluid resuscitation under sepsis protocol was administered; however, the blood pressure remained low, warranting initiation of intravenous norepinephrine titrated to 5-15 mcg/minute and admission to the intensive care unit (ICU). The patient's hypotension initially worsened due to attempts at rate control, and it remained persistent despite eventual control of the heart rate. Antibiotics were transitioned to IV levofloxacin 750 mg daily and IV metronidazole 500 mg every six hours on arrival to the ICU. Over the next 24 hours, the patient's condition rapidly deteriorated, evidenced by increased vasopressor requirements, the elevation of fever to 103.9 °F, lactic acid rising to 8.1 mmol/L, WBC increasing to 34,900 K/uL, and the worsening of renal function.

Multiple specialties were consulted, including infectious diseases, vascular surgery, orthopedic surgery, podiatry, wound care, and nephrology. Preliminary blood cultures demonstrated gram-negative bacilli, warranting the escalation of antibiotics to meropenem 500 mg every six hours by infectious diseases. Given the severity of the patient's lower extremity wounds, the initial source of infection was thought to be likely lower extremity cellulitis although left knee septic arthritis was considered as well. Due to the patient's rapid clinical decompensation and increasing swelling, erythema, and lack of mobility of the left knee and the presence of an effusion in the right knee, a bilateral knee arthrocentesis with fluid studies was performed on hospital day two with evidence of purulent fluid encountered on left knee aspiration. Further speciation of initial blood cultures unexpectedly demonstrated *P. multocida* (2/2 cultures). Given the patient's history of beta-lactam allergy, meropenem 500 mg every six hours was continued at the recommendation of infectious diseases. On further questioning, the patient reported having four pet cats at home, but adamantly denied any recent history of traumatic bites, scratches, or exposure of chronic lower extremity wounds to cat saliva. Further history was obtained from the patient's wife, who was also adamant that the patient had not had any invasive exposure to the pet cats at home. She denied him ever sleeping in the same bed as the cats, or lying on the floor and playing with the pets, and confirmed that the patient's lower extremity wounds had not been exposed to cat saliva in the recent past.

Fluid analysis from left knee aspiration revealed a WBC count of 396,250 cells/uL, consistent with septic arthritis, while fluid analysis from right knee aspiration was benign. The patient was deemed too unstable to be taken to the operating room for incision and debridement of the left knee given his ongoing vasopressor requirements. Instead, serial left knee aspirations were performed with repeated fluid studies on hospital day six, demonstrating fluid WBC of 136,355 cells/uL, an improvement from earlier levels but still notably elevated. Fluid cultures from the initial left knee aspiration did not grow any bacterial organisms; however, this could be attributed to the fact that aspiration was performed after the patient had received multiple doses of several different IV antibiotics. The patient clinically improved and was transferred out of the ICU and taken to the operating room on hospital day eight for incision, debridement, and washout of the left knee. Upon entering the knee joint, copious amounts of purulent material were found with extension into the patellofemoral joint. Intraoperative aerobic/anaerobic cultures were obtained but did not grow any organisms. The patient continued to have persistent left knee effusion despite aggressive incision and debridement.

On hospital day 16, the patient continued to complain of severe left knee pain and swelling. Given the persistent left knee effusion, the patient was taken back to the operating room for a repeat left knee arthrotomy with irrigation and debridement. The left knee continued to have purulence with extension into the subcutaneous tissue, warranting copious irrigation, debridement, and the placement of an intraarticular Hemovac drain. The patient continued to improve clinically, and repeat blood cultures returned negative. Multiple finalized cultures from the left knee did not show bacterial growth. The patient had a prolonged hospital course complicated by acute renal failure, electrolyte abnormalities, elevated transaminases, and acute exacerbation of chronic congestive heart failure. He was eventually discharged in stable condition to a long-term acute care facility with plans to complete a six-week course of IV meropenem 500 mg every six hours.

## Discussion

Sepsis is one of the most common causes of hospital admissions, accounting for more than 640 admissions per 100,000 people, and it remains one of the most common causes of death among critically ill patients [1]. Not only is sepsis challenging for patients, but it is very challenging for hospital systems as well, as the cost of medical management associated with the condition ranks among the highest for all admission diagnoses, with some estimates noting upwards of $20 billion in yearly hospital expenses for these patients. While cases of gram-positive infections are on the rise, gram-negative bacteria remain the most common cause of septicemia requiring hospitalization. Among specific organisms, *Staphylococcus aureus*, *Pseudomonas*, and Enterobacteriaceae are the most common pathogens in patients presenting with sepsis [2]. Regardless of the associated pathogen, it has commonly been reported that the lungs are most often the primary site of infections, followed by the abdomen and genitourinary tract [3]. While sepsis secondary to skin and soft tissue infections is not uncommon, it occurs less frequently than sepsis related to other major organ systems. Hence, our patient's case with *P. multocida* bacteremia and left knee septic arthritis was a unique outlier among the commonly observed epidemiologic trends.

*P. multocida* is a gram-negative coccobacilli, which is commonly found within the respiratory tract and gastrointestinal flora of many animals. It is also generally associated with human transmission from cat and dog bites. Other animals such as rats, horses, and rabbits have also been implicated in *P. multocida* infection. Penetrating injury is the most common mechanism of entry into the body; however, there have been several reported cases of *P. multocida* infections secondary to non-traumatic exposure to animal secretions as well [4]. Skin and soft tissue infections are the most common manifestation of *P. multocida* infections in humans. Presentations can vary, ranging from local abscess formation and tenosynovitis to more serious cases including septic arthritis or osteomyelitis, generally depending on the depth of traumatic injury and inoculum present [4]. Respiratory tract infection secondary to *P. multocida* has also been reported; however, in such cases, *P. multocida* is seen as a commensal organism, often affecting patients with underlying lung disease. Other systemic infections have also been reported, including meningitis, brain abscesses, spontaneous bacterial peritonitis, and intra-abdominal abscesses [5]. Bacteremia in itself is a very rare presentation of this pathogen, with one retrospective study reporting an incidence of 11% in a population of 952 patients with known *P. multocida* infection [6]. In general, *P. multocida* is an opportunistic pathogen with a higher incidence in patients with immunocompromised states, such as diabetes, HIV, advanced liver disease, and chronic kidney disease [5]. While our patient did own four cats, both he and his wife were adamant that he had not been recently bitten or licked by their pets. His poorly controlled diabetes and chronic lower extremity wounds served as independent risk factors for severe infection. Patients with diabetic foot infections and chronic lower extremity ulcerations should be counseled frequently about the avoidance of pets at home in order to avoid exposure to infectious pathogens. In such cases, a home visit would likely help to eliminate risky behaviors, which could help avoid further complications.

In our patient, *P. multocida* was likely introduced through his chronic lower extremity lesions. It is very possible that his pet cats introduced the organism through their saliva, seeding the circulatory system and eventually spreading hematogenously to his left knee. While intraoperative cultures did not grow any organisms, this could be explained by the fact that the patient had already received significant intravenous antibiotic therapy in the setting of his critical illness. Given the rarity of *P. Multocida* bacteremia, hematogenous spread with subsequent development of septic arthritis is worthy of documentation and further investigation in the future, as multiple challenges usually arise in the treatment of these patients. While penicillins are the mainstay of therapy, due to our patient’s beta-lactam allergy, he was started on meropenem and had a positive response. In general, *P. multocida* bacteremia carries a high fatality rate of about 30% despite aggressive treatment [7]. Despite evidence of life-threatening septic shock, our patient did remarkably well considering the circumstances.

## Conclusions

While *P. multocida* is a common pathogen associated with skin and soft tissue infections, bacteremia secondary to this organism is rare and is associated with high mortality rates. We discussed the case of a chronically ill patient who presented with septic shock secondary to *P. multocida* bacteremia with findings of left knee septic arthritis from hematogenous seeding, despite the adamant denial of close contact with any animals. This case emphasizes the need for clinicians to review the risk factors for infections with patients, especially those with comorbid conditions that predispose them to immunocompromised states. Additionally, this case documents a unique presentation of *P. multocida* bacteremia that was treated successfully despite a reported beta-lactam allergy, providing valuable information for clinicians encountering similar challenging cases in the future.
